# Imaging subsurface structures near wells in the northwest Geysers geothermal site

**DOI:** 10.1038/s41598-026-57720-x

**Published:** 2026-06-15

**Authors:** Taghi Shirzad, Mohsen Kazemnia, Beata Orlecka‐Sikora, Götz Bokelmann, Saman A. Aryana

**Affiliations:** 1 Institute of Astronomy, Geophysics and Atmospheric Sciences, University of São Paulo, São Paulo, 05508-090 Brazil; 2 Department of Meteorology and Geophysics, University of Vienna, Vienna, Austria; 3 Institute of Geophysics, Polish Academy of Sciences, 01-452 Warsaw, Poland; 4 Department of Chemical and Biomedical Engineering, University of Wyoming, Wyoming, USA

**Keywords:** Micro-earthquake seismicity, Surface wave tomography, Inter-event interferometry, The Geysers Geothermal site, Energy science and technology, Solid Earth sciences

## Abstract

Vapor-dominated geothermal systems provide a reliable, low-carbon source of heat and electricity, but optimizing their exploitation requires high-resolution imaging of fracture networks and fluid pathways at the reservoir scale. We analyze a dense microseismic cluster in the northwestern Geysers (California), selecting 1,276 induced earthquakes recorded between 2006 and 2015. Using inter-event interferometry and fast-marching surface-wave tomography, we retrieve Rayleigh wave phase velocities on horizontal layers at 100 m spacing and jointly invert them with previously derived local group velocities to obtain a quasi-3D shear-wave (Vs) model at ~ 100 × 100 × 10 m blocks. The resulting Vs models reveal three main types of low-velocity anomalies: (I) fault-related zones associated with fracturing and hydrothermal alteration, (II) shallow steam-cap and normal-temperature-reservoir (NTR) boundary transitions spanning depths of ~ 900–1400 m, exhibiting sharp Vs contrasts due to thermal and fluid effects, and (III) injection/engineering-related anomalies characterized by localized or vertically elongated low Vs patches. Integrating these results with induced microseismicity provides valuable insights into fracture activity, fluid migration, and stress evolution within the geothermal reservoir.

**Supplementary Information:**

The online version contains supplementary material available at https://doi.org/10.1038/s41598-026-57720-x.

## Introduction

Geothermal energy is a reliable low-carbon alternative to fossil fuels, yet its exploitation requires accurate characterization of subsurface fluid phases, heat sources, and fracture networks. A geothermal system forms where heat from the Earth’s interior is sufficiently concentrated to be economically extractable, typically above shallow intrusions or anomalously high geothermal gradients and hosted in permeable lithologies that can store and transmit hot fluids. Geothermal reservoirs are commonly classified by reservoir temperature as low-enthalpy (< 150 °C), medium-enthalpy (150–200 °C), or high-enthalpy (> 200 °C), following the exergy-based scheme of^1^. This classification guides exploration strategy, particularly because geophysical responses depend strongly on the dominant fluid phase (liquid versus vapor). While most fields are liquid-dominated, vapor-dominated systems are rare, notable examples being Larderello (Italy), Matsukawa (Japan), and The Geysers (USA)^2^.

The Geysers in northern California is the world’s largest vapor-dominated geothermal system and a unique site for electricity production. In its northwestern sector, overall reservoir temperatures range from approximately 240–340 °C (see^2^), with temperatures at depth approaching or exceeding 400 °C in the deepest portions of the EGS zone^3^, classifying the system as high-enthalpy. Heat is supplied by cooling granitic intrusions, and steam accumulates in a highly fractured rock sequence capped by low-permeability clay^2–4^. Seismically, zones of intense fracturing and partial steam saturation display low shear-wave velocity (Vs) and high levels of induced seismicity^5^. Hydrothermal alteration can further modify Vs by weakening rock fabrics and increasing porosity through clay formation. At The Geysers, this process predominantly lowers velocities^6^. The reservoir is primarily steam-filled, with fractures and pore spaces occupied by superheated vapor^7^. Since the late 1990s, injection has been used to sustain steam production^3^, but has also coincided with a measurable increase in seismic activity^8^. Seismological approaches have been widely applied to characterize reservoir structure, stress, and fluid-induced variations at The Geysers, including the effects of fluid injection^8,9^, temporal variations in seismicity^9,10^, and earthquake source mechanics^9–12^.

Building on this context, the development of an Enhanced Geothermal System (EGS) development at The Geysers since 2008 further altered the seismic response, with focal mechanism analyses of larger-magnitude events revealing an increased proportion of reverse faulting solutions,particularly near low Vp/Vs anomalies^5^. Most seismicity occurs within the upper 5 km of crust^7^ due to thermoelastic fracturing, pore-pressure diffusion, and stress redistribution. Shallow seismicity (< 1 km) is generally consistent with the regional tectonic stress field, expressed predominantly as strike-slip and normal faulting mechanisms^6,13^, while deeper reservoir seismicity is additionally influenced by local stress perturbations from fluid injection and thermal effects^6,14^. Shear-wave splitting studies reveal vertically oriented fractures with fast directions aligned to maximum horizontal stress (N23°E), consistent with normal and strike-slip faulting^6,14^. Orientations vary between N–N60°E in the northwest and N5°E–N85°E in the south^15^, providing critical constraints for tracking fluid pathways and seismicity patterns.

Seismic tomography confirms that the main steam field is characterized by low Vp, Vs, and Vp/Vs^5^. Over 260,000 seismic events recorded since 1984 provide an exceptional dataset for imaging^16^. Microseismic analyses reveal temporal variations in hypocenter distribution, b-values, and **M**_W_ ≥ 4 event rates^8^. However, uncertainties in velocity models limit raypath estimation and Green’s function computation^13^. To address these issues, 3D P- and S-wave velocity models from traveltime tomography have been incorporated into waveform inversions^17^.

Despite these advances, conventional body wave tomographic techniques (e.g.^5,18^) and ambient noise surface wave tomography (e.g.^19–22^) often lack sufficient spatial resolution at the depth range relevant to geothermal reservoirs, where station spacing, path coverage, and usable frequency content collectively constrain the minimum resolvable feature size. We note that some ambient noise approaches targeting shallower depths with higher frequency content have achieved sub-kilometer resolution, but this has not been demonstrated at the reservoir depths of interest here. Similarly, Vp/Vs ratio monitoring (e.g.^17^) tracks temporal changes without producing full spatial images. To overcome these limitations, seismic interferometry has been developed to retrieve empirical Green’s functions (EGFs) by cross-correlating event pair recording on a common station^23^. This inter-event interferometry provides higher-resolution imaging by exploiting dense earthquake clusters to improve path coverage^23,24^. In this study, we extend inter-event interferometry to extract Rayleigh-wave phase velocities from vertical-component waveforms in a dense seismic cluster in the northwestern Geysers. Combined with previously measured group velocities^25^, these data yield a high-resolution quasi-3D Vs model. Our results provide new insights into reservoir lithology, stress regimes, and fluid pathways, which are key parameters for geothermal resource management and seismic hazard mitigation.

## Data and methods

**Figure 1:**
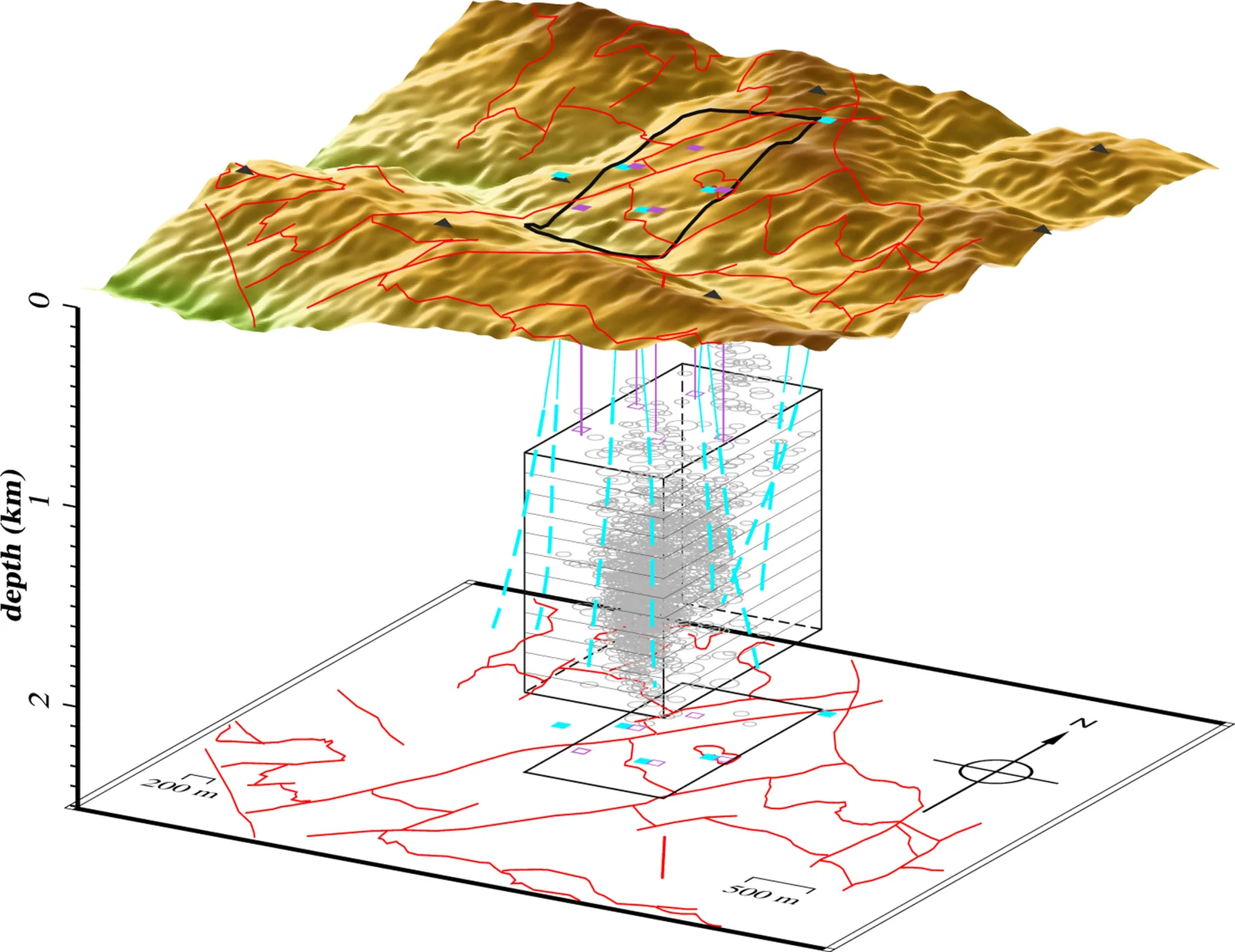
The study area (cuboid) in the NW part of The Geysers geothermal field, California, USA. The projected study area appears as a rectangle on the topographic map (top) and horizontal map (bottom). Injection and steam wells are shown by blue and purple lines, respectively, with wells located inside the cuboid indicated by dashed lines^48^. Major fault zones (red lines) and induced seismic events (gray circles) are also displayed. Projected wells on the topographic map, cuboid, and horizontal map are marked by squares in corresponding colors. This figure depicted by GMT version 6.4.0 (^47^; https://www.generic-mapping-tools.org), the known faults are from^49^.

We used an earthquake catalogue from the IS-EPOS platform (European Plate Observing System, Thematic Core Service on Anthropogenic Hazards), specifically the Geysers Prati-9 and Prati-29 cluster episode^26,27^. The dataset comprises 1,276 micro-earthquakes with the coda duration magnitudes (**M**_d_) between 0.95 and 3.16. These events occurred from January 2006 to June 2015 within a volume approximately 1 × 2 km^2^ in lateral extent and 0.9–2.2 km in depth (Fig. 1), located predominantly in the high-temperature reservoir zone between the injection wells Prati-9 and Prati-29^28^. All selected events were recorded by at least 17 stations and meet strict quality criteria including: (1) hypocentral uncertainties < 50 m, (2) azimuthal gaps < 75°, and (3) root-mean-square, RMS, location residuals < 0.05 s^26,29^.

Continuous waveform data were retrieved from the NCEDC^16^ and subjected to automated quality control to remove records with instrumental artifacts, gaps, or excessive technical noise (RMS ≥ 2). Only vertical-component (Z) seismograms with high signal-to-noise ratios (SNR ≥ 4) were retained. Following^30^, SNR was defined as the ratio of the maximum envelope amplitude within the expected Rayleigh-wave window to the RMS noise level in a pre-signal window. Each selected trace was detrended, mean removed, and corrected for instrument response. We then applied one-bit normalization^31^ and spectral whitening to suppress amplitude variations and enhance phase coherence in cross-correlations. Although this processing eliminates physical amplitude information, it does not affect the analysis since we rely exclusively on phase-derived measurements.

EGF signals were derived by cross-correlating preprocessed waveforms for all event pairs^23^. Pairs were retained when the azimuths from both events to a common station differed by ≤ 1°, approximating a stationary-phase geometry^32^. For pairs recorded at multiple common stations, cross-correlated functions were stacked using the phase-weighted stacking (PWS) method^33^. Before stacking, energy outside the expected Rayleigh-wave window, which contains body-wave arrivals, was muted to zero^25^, and the remaining segments were tapered with a Hann window to minimize edge effects^34^.

**Figure 2:**
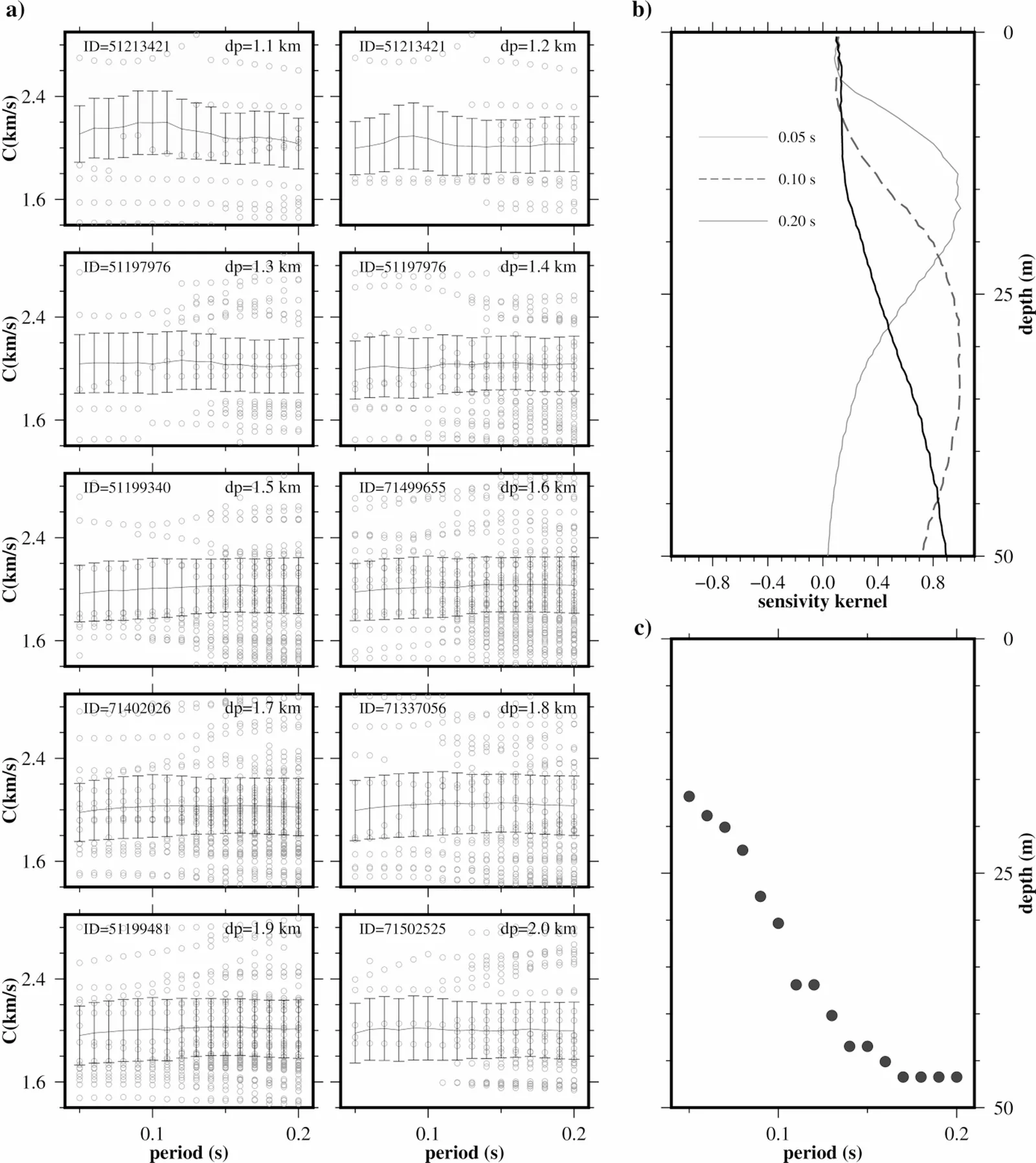
(**a**) Gray circles show individual Rayleigh-wave phase-velocity dispersion curves for all EGF pairs in which the event identified by the shown ID participates, plotted separately by event to avoid saturation of the figure. The thin black line is the average phase velocity dispersion curve, C_0_, computed from the full set of all available dispersion curves at that depth layer (*dp*). For each period, the error bar on C_0_ shows the ± 3σ range used as the data-selection criterion for the tomographic inversion; only measurements within this range are retained. (**b**) Normalized Rayleigh wave phase velocity sensitivity kernels for periods of 0.05, 0.1, and 0.2 s represent the partial derivative of phase velocity with respect to Vs; sensitivity to Vp and density is negligible at these periods and is not shown. (**c**) Solid black circles denote the maximum depth of the sensitivity kernel functions for period range of 0.05–0.2 s.

**Figure 3:**
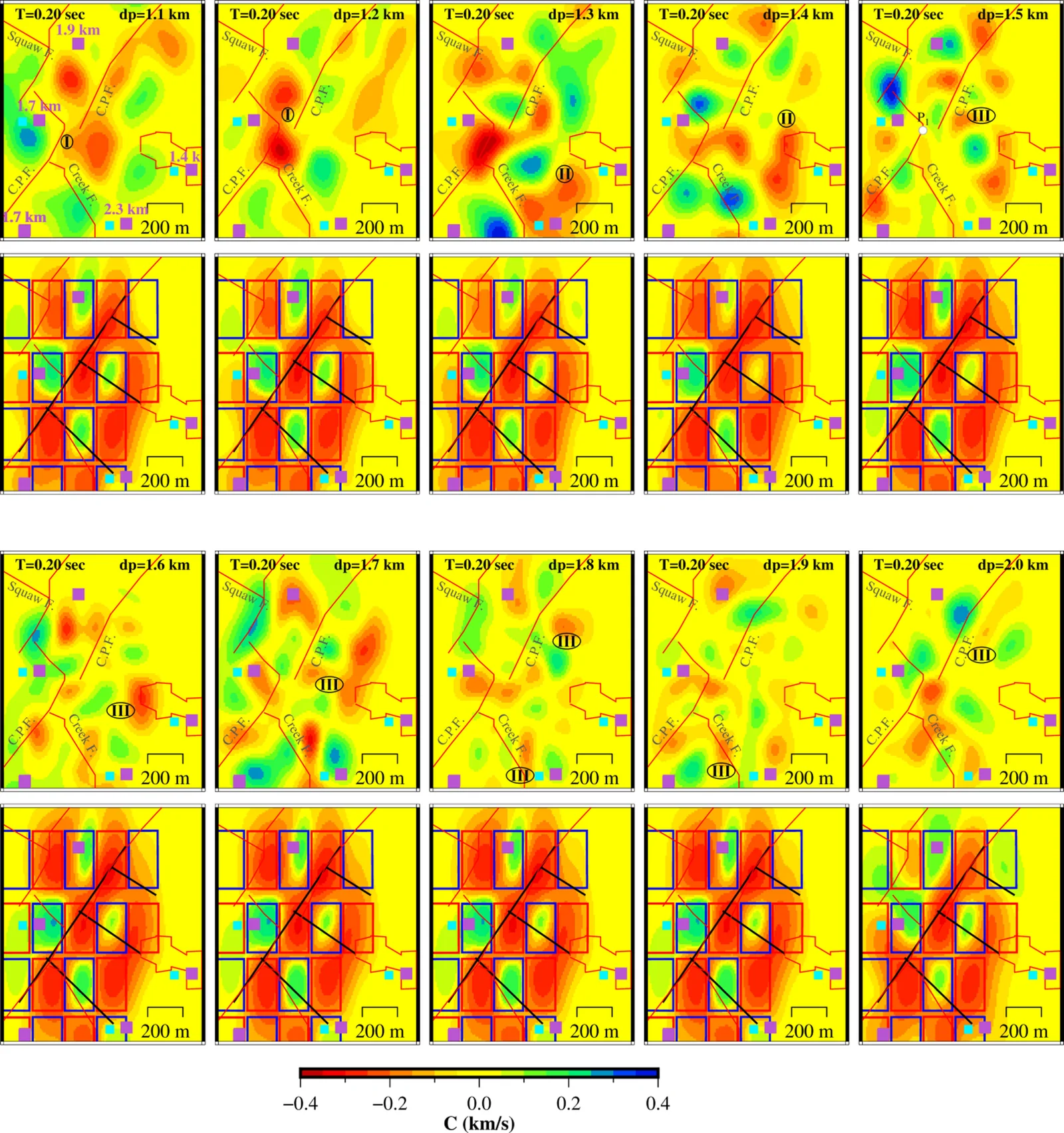
Selected depth slices of the phase velocity tomographic maps and corresponding checkerboard resolution test results. The marked low-velocity anomalies on tomographic maps are associated with (I) fault systems, (II) variations in the topography of the normal temperature reservoir (NTR topography^9^), and (III) injection/engineering-related anomalies (see text for details). The alternative low (red box) and high (blue box) velocity anomalies in checkerboard are *C*_*0*_ ± *0.3* km/s. Known faults (Squaw Fault, Creek Fault, and Caldwell Pines Fault, C.P.F.) are shown as red lines. Projected surface/well-head locations are shown as blue squares and steam wells as purple squares, consistent with Fig. 1. The circle at depth of *dp* = 1.5 km indicates grid point P_1_ in Fig. 4. The black thick lines in checkerboard results are shown the profiles in Fig. 6.

Following^25^, each EGF within ± 50 m of a reference layer was projected onto layers spaced every 100 m (thin layers in cuboid of Fig. 1). Dispersion curves for each layer were measured using the spectral method of^35^, which applies Aki’s formulation by matching zeros in the observed spectrum to zeros of the Bessel function. Phase velocities were estimated at discrete periods of 0.05–0.20 s. The light gray circles in Fig. 2a show selected phase velocity dispersion measurements for each depth layer. These measurements were then inverted using fast marching surface wave tomography (FMST^36^) to obtain 2D phase velocity maps for successive layers, proceeding from top to bottom as described by^25^. The tomography employed a 100 × 100 m horizontal grid, using the average phase velocity at each period as the starting model (*C*_*0*_; thin lines in Fig. 2a) and optimum regularization parameters determined via L-curve analysis. The average dispersion curve, *C*_*0*_, is computed from all quality-controlled measurements, with outliers removed using a 3σ criterion. Moreover, dispersion curves within the *C*_*0*_ ± *3σ* range, where *σ* is the standard deviation of phase velocities (bars in Fig. 2a), were included in the tomographic inversion. Figures 3 and S1 show representative tomographic maps at various depths.

Before further processing, the reliability of the tomographic results should be assessed. The checkerboard resolution test is a fundamental procedure in Rayleigh wave phase velocity tomography, as it evaluates the model’s ability to resolve spatial variations. By comparing synthetic input anomalies with their recovered counterparts, this test provides critical insights into the robustness and spatial resolution of the tomographic model. The alternating low and high velocity anomalies, *C*_*0*_ ± *0.3* km/s, are set to twice the horizontal cell size to suppress any leakage of resolvable structures by the data^37^. However, the inversion parameters (e.g., regularization, inversion cell size, starting model, etc.) are kept identical to those applied in the inversion of the observed data. Figure 3 shows the recovered checkerboard test resolution maps.

**Figure 4:**
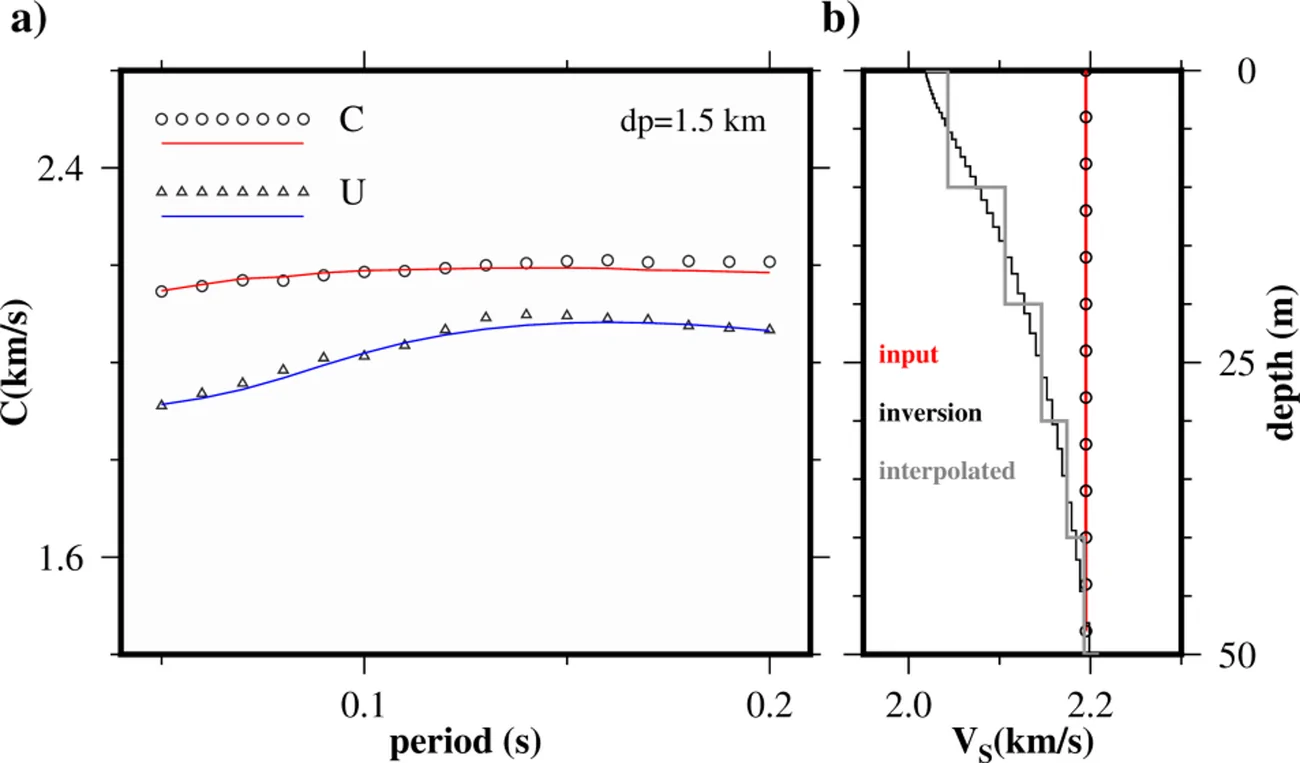
(**a**) Local group, *U* (triangles), and phase, *C* (circles), dispersion curves at grid point P_1_ (see Fig. 3, *dp* = 1.5 km) for the depth layer of *dp* = 1.5 km . Group-velocity data are from^25^. Solid blue and red lines represent the inverted dispersion curves from the output model in (**b**) for group and phase measurements, respectively. (**b**) Input Vs model (red) parameterized at 4 m depth intervals (black open circles). The inversion result (incorporating both layer thickness and Vs) is shown as the black 1D Vs model at grid point P_1_. The resampled 1D Vs model to a uniform 10 m depth grid, is shown in gray.

**Figure 5:**
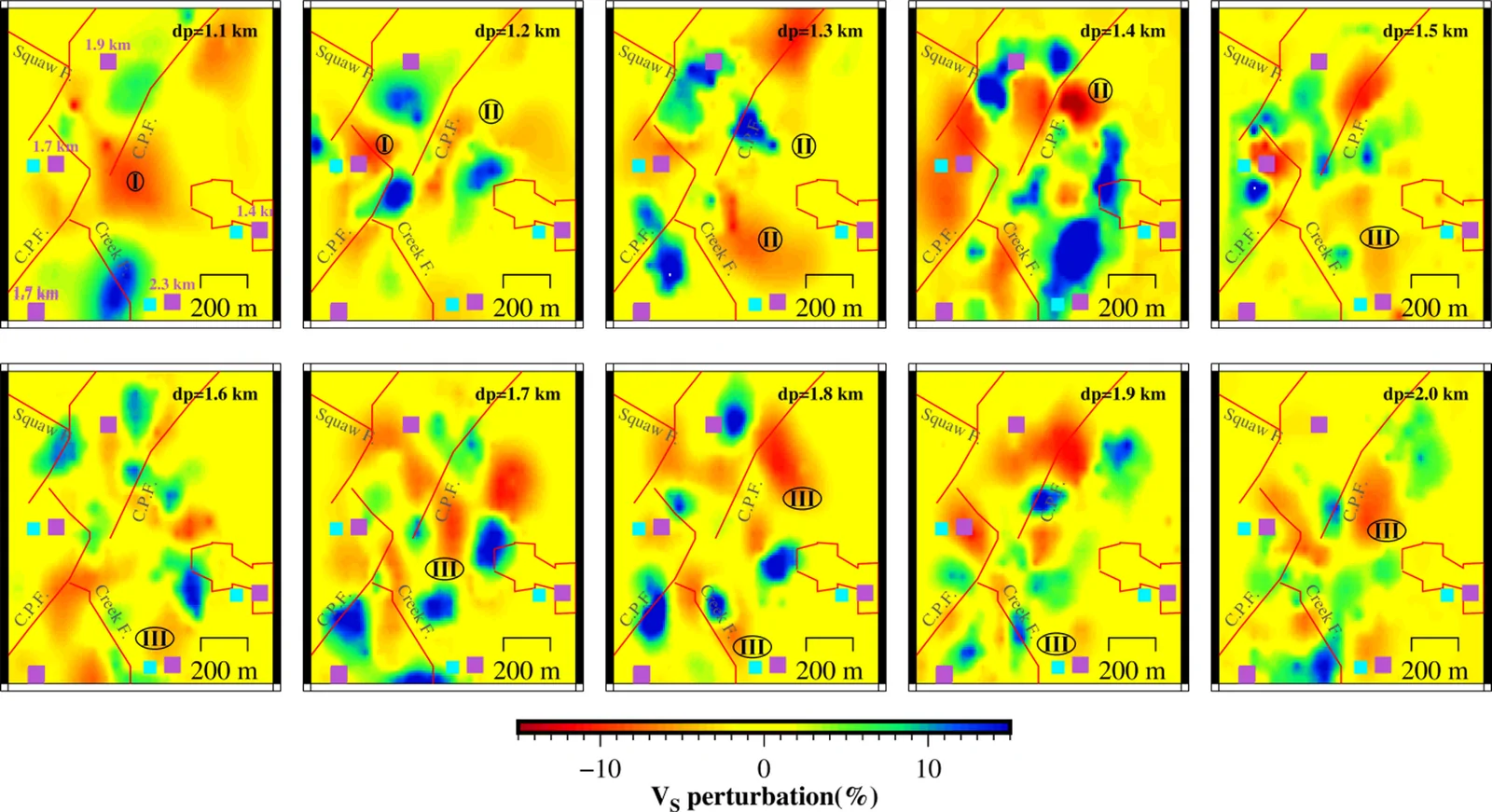
Selected horizontal maps of Vs perturbation models. Distinct low-velocity anomalies highlight (I) fault-related structures, (II) variations in NTR topography, and (III) injection/engineering-related anomalies (see text for discussion) Major faults, including the Squaw Fault, Creek Fault, and Caldwell Pines Fault (C.P.F.), are shown as red lines. Projected surface/well-head locations are depicted as blue squares and steam wells as purple squares, consistent with the symbology as colors used in Fig. 1.

Finally, for each individual layer, local Rayleigh wave phase dispersion curves, *C(T)*, at each tomographic grid point, and group velocities (extracted from the previous study^25^), *U(T)*, were simultaneously supplied as independent datasets as input to the 1D Vs inversion. We applied the iterative damped least-squares algorithm, as implemented in *surf96*^38^, to obtain the 1D Vs model for each local dispersion curve in each layer. Figure 4a shows a local dispersion curve at grid point P_1_ (see triangles and circles in Fig. 3) for depth layer of *dp* = 1.5 km. The initial 1D Vs model (red model in Fig. 4b) for inversion was derived from a previously published 1D Vp model for The Geysers^5^ using a uniform Vp/Vs ratio of 1.7^5,17^. Based on the sensitivity kernel functions (Fig. 2b,c), the effective depth of each period (defined as the depth at which the kernel amplitude drops to approximately 30% of its maximum) increases with period, ranging from about 8 m at 0.05 s to about 48 m at 0.20 s. This means that the complete set of phase velocity measurements used in this study samples Vs structure over a depth range of approximately 8 to 48 m (see black circles on red model in Fig. 4b). The regularization parameter was selected using the L-curve method to balance the trade-off between data misfit and model smoothness. Figure 4b shows the calculated 1D Vs model at grid point P_1_ (see black model), which can be inserted at depth *dp* ± 50 m to fill the 100 m depth interval gap between layers. Because the 1D inversion was performed for both layer thickness and Vs, the calculated 1D Vs model was resampled to construct the final model with uniformly 10 m interval layers (gray model in Fig. 4b). Repeating this procedure across all grid point of layers generated the quasi-3D Vs model. Figure 5 shows horizontal slices of calculated Vs models.

## Results and discussion

Although the virtual seismometer technique is, in principle, applicable across a wide range of spatial scales, most prior applications have focused on regional studies (e.g.^32^, with 30 km × 30 km inversion cells) or on localized settings (e.g.^39^, with 1.1 km × 1.1 km cells). When adapted to the scale of a site (e.g., geothermal systems), however, this framework becomes a powerful tool for imaging subsurface heterogeneity at depths directly relevant to site exploitation, particularly in areas affected by fluid injection or infiltration and consequently induced seismicity. The tomographic images obtained in such settings not only enhance structural characterization of geothermal reservoirs but also provide valuable insights for evaluating reservoir quality, optimizing exploration strategies, and reducing uncertainties associated with sustainable resource development.

In the present study, we extend the method to finer resolution (100 × 100 × 10 m cells) to resolve the 3D seismic structure of small-scale targets, exemplified by the injection and steam wells in the northwestern Geysers geothermal site. For periods of 0.05–0.2 s, depth-dependent tomographic models were generated by computing differential phase velocity maps between successive layers defined by vertical intervals. Interval thicknesses were selected following strict criteria from earlier studies (e.g.^25^), considering the number and distribution of seismic events and uncertainties in hypocentral locations. Although our quasi-3D Vs model captures key structural and operational features, some limitations must be acknowledged. The contrasting anomalies between *C* and *U* tomographic maps (e.g.^25^), even after sensitivity normalization, may arise from fundamental differences in how these measurements interact with subsurface structures. Scattering from small-scale heterogeneities, waveform distortion due to anelastic attenuation from fluid infiltration, data noise and resolution trade-offs, and artifacts from inversion regularization all contribute to these discrepancies. Some of these issues (such as inversion artifacts or noise-resolution trade-offs) can be mitigated through synthetic tests (e.g., checkerboard test resolution, etc.), whereas others, notably scattering and attenuation, require further investigation, with low velocities linked to fluid infiltration clearly observed around well locations. Moreover, apparent phase velocities can be biased by attenuation effects, which alter dispersion differently for group and phase velocities, suggesting that part of the *C-U* discrepancy may reflect frequency-dependent attenuation rather than purely elastic structure. In addition, virtual-source geometry and noise distribution can introduce directional sensitivity. To quantify these effects and assess robustness, in addition to synthetic resolution analyses, future work should incorporate time-lapse imaging to distinguish structural changes from processing artifacts.

**Figure 6:**
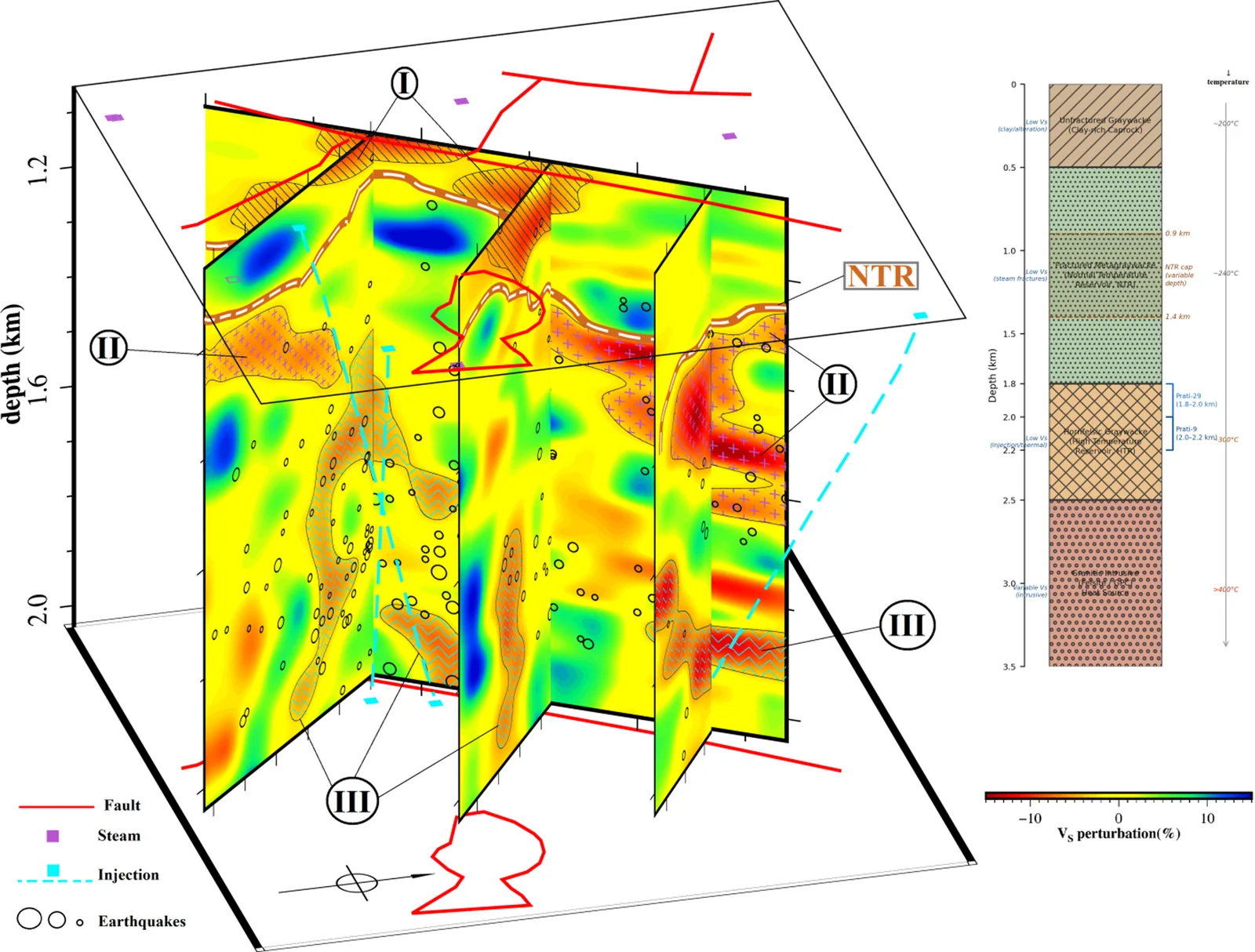
Vs perturbations along four profiles, parallel to the C.P.F. and perpendicular sections crossing the three main low-velocity anomalies. Anomalies are contoured where Vs ≤ –5%. Known faults and wells are projected onto the top and bottom faces of the cuboid. Injection wells are shown as blue dashed lines. Black circles mark hypocenters located within ± 25 m of each profile^26,50^. The NTR (from^9^) is outlined by a thick brown solid line and a surrounding dashed white line. Anomaly types are (*I*) fault-related fracture zones and hydrothermal alteration, (*II*) steam-cap and NTR-topography transitions, and (*III*) injection/engineering-related anomalies at or above injection well depths. Well traces are plotted to their true total drilled depths. Horizontal markers indicate the top and bottom depths of the well segments located within the study volume. One injection well has its wellhead outside the cuboid but is completed at ~ 1.8 km depth within the modeled region; its termination depth is plotted correctly. The inset panel (right) shows a schematic stratigraphic column for the NW Geysers study volume, synthesized from^2,3,9^, with the four main lithological units (clay-rich caprock, fractured metagraywacke/NTR, hornfelsic graywacke/HTR, and granitic intrusive/felsite), their approximate depth ranges, and the corresponding Vs behavior expected for each unit. This figure depicted by GMT version 6.4.0 (^47^; https://www.generic-mapping-tools.org).

Based on the 2D tomographic (Figs. 3, S1) and obtained Vs maps (Fig. 5), the observed low-velocity anomalies can be grouped into three categories: (*I*) those associated with fault structures, (*II*) those influenced by topographic variations at the steam boundary between two layers, and (*III*) those related to fluid injection or infiltration. Low velocity zones associated with fault structures likely reflect fracturing, brecciation, and hydrothermal alteration along fault planes. Fault zones typically consist of crushed, fluid-filled rock with reduced elastic moduli, resulting in lower seismic velocities^40^. The enhanced porosity and permeability^41^ along these structures facilitate the circulation of hydrothermal fluids and gases, which further modify rock properties^42^ through mineral dissolution, clay formation, and precipitation of secondary minerals (e.g., silica, calcite). The first category (I) anomalies typically appear as pronounced impedance contrasts on tomographic maps^43^, causing scattering and diffraction of seismic waves; nevertheless, they do not produce a continuous low-Vs signature along all fault traces, a pattern that more plausibly reflects the inherent heterogeneity of fault damage zones than any weakness in the interpretation. Damage is most intense at structural complexities such as fault intersections and segment boundaries, and in Fig. 6, the strongest type-I patches are spatially coincident with the projected locations of such features. This segmented spatial pattern is consistent with field and borehole observations of fault damage at analogous hydrothermal settings^40,41^. The heterogeneity of fault damage zones explains the persistent low Vs anomalies observed across various periods/depths in Figs. 3, 5, 6, and S1.

The second group of anomalies (II) is distributed across the depth range spanned by the NTR surface, which varies laterally from approximately 900–1400 m across the study area^3,9^, and their spatial pattern traces the topographic relief of this boundary rather than forming a continuous horizontal layer. Where the NTR surface dips or shallows over short lateral distances, the transition between steam-saturated rock above and liquid-saturated rock below occurs at markedly different depths within the study volume. This creates abrupt lateral Vs contrasts at any given depth slice, as some grid points sample steam-dominated rock while adjacent points at the same depth sample the cooler, water-saturated formation below the cap^3,9^. The irregular NTR topography thus produces a patchwork of low-Vs zones that mirrors the undulating steam-boundary geometry rather than forming a continuous horizontal layer. In brief, at the regional scale, the NTR surface is relatively smooth, but at shorter wavelengths it may exhibit small-scale undulations and protrusions generated or amplified by pre-existing structural damage and lithological heterogeneity, producing a locally irregular boundary geometry. The sharp Vs transitions highlighted in Fig. 6 directly reflect this geometry: the NTR boundary (outlined by a thick brown line) separates low-Vs steam-cap material above from relatively higher-Vs material below, and the lateral positions of these transitions track the local dip and shape of the NTR surface. Lithological heterogeneity within the cap zone (e.g., interbedded volcanics, altered tuffs, and sediments^3^; adds secondary complexity to the velocity pattern, but the primary control on the spatial distribution of type-II anomalies is the topographic character of the steam boundary itself.

Moving from structural to operational controls, the third category of anomalies, (*III*), is strongly influenced by engineering activities, particularly fluid injection into geothermal reservoirs. Injection introduces cold water into hot, fractured host rocks, producing a range of effects: (1) thermal contraction and cracking of rock^44^, (2) pressure-driven fluid migration^45^, and (3) localized mineral dissolution/precipitation^46^. These processes alter elastic properties and pore structure, leading to pronounced low Vs patches at or above injection depths of Prati-9 (~ 2.0–2.2 km) and Prati-29 (~ 1.8–2.0 km), as documented in^3,9^. It should be noted that both wells are deviated from vertical axis (see Fig. 1), so their subsurface injection points are laterally offset from the well-head symbols shown in Figs. 3 and 5, which represent projected surface locations only. The actual spatial footprint of injection-induced velocity perturbations therefore extends well beyond the immediate wellbore and cannot be read directly from the well symbols in a given depth slice.

The lateral extent of this influence zone is depth-dependent and controlled by the local permeability and fracture architecture. In highly permeable rocks (e.g., fractured greywackes and altered volcaniclastics that dominate the primary injection interval between approximately 1.5 and 2.5 km depth), injected fluids spread laterally along the dominant fracture network. Based on seismicity cloud dimensions and hydraulic diffusivity, the lateral radius of influence on Vs reaches up to 500 m at the main injection interval. Because the NW Geysers reservoir is highly fractured and mechanically anisotropic, this zone of influence is not a uniform cylinder around the well but an elongated ellipsoid, typically 20–30% wider along the direction of maximum horizontal stress (N–NE^6^;), consistent with the observed spatial distribution of type-III low-Vs patches across Figs. 3, 5, and 6. In addition, highly permeable units such as fractured greywackes and metagreywackes that dominate the reservoir between approximately 1.2 and 2.0 km depth (see stratigraphic inset in Fig. 6^2,3^;) provide preferential vertical pathways for fluid migration. In these intervals, buoyant rise of injected fluids along connected fractures may generate vertically elongated low-Vs anomalies that extend beyond the primary injection depth. The spatial correspondence between the type-III low-Vs patches and this lithological interval in Fig. 6 supports a permeability-controlled migration mechanism.

At shallower depths, where pore pressure decreases and rock permeability is lower, the radius contracts up to 200 m and anomalies remain localized and patchy rather than laterally continuous. In contrast, where fluid can rise buoyantly along permeable pathways, vertically extended low-Vs anomalies develop, spanning depth intervals greater than the injection zone itself. Moreover, in less permeable rock, this lateral spread is restricted, and anomalies remain confined to individual fracture corridors. The temporal variability of such anomalies (linked to injection cycles and well activity) makes them particularly diagnostic of reservoir dynamics. Although the present study does not resolve time-lapse Vs variations, the spatial patterns of type-III anomalies are consistent with injection-driven velocity changes documented at The Geysers using coda wave interferometry^19,20^ and Vp/Vs ratio monitoring^17^. The dependence of anomaly amplitude on permeability and fracture connectivity^41^ underscores the diagnostic potential of time-resolved velocity monitoring. Future application of inter-event interferometry to successive time windows, as discussed in^25^, could directly track the temporal evolution of these anomalies, distinguish structural changes from processing artifacts, and provide complementary constraints on injection-driven fracture activation and fluid migration.

## Conclusion

This study demonstrates the effectiveness of inter-event interferometry combined with high-resolution Rayleigh-wave tomography for imaging quasi-3D Vs structure in the northwestern Geysers, the world’s largest vapor-dominated geothermal field. The resulting model, resolved at a site-relevant scale of approximately 100 × 100 × 10 m, reveals three primary classes of low Vs anomalies: (*I*) fault-controlled zones indicative of fracture damage and hydrothermal alteration, (*II*) steam-cap/topographic transitions at ~ 0.9–1.4 km depth associated with sharp thermal and fluid contrasts, and (*III*) injection- and engineering-related anomalies linked to thermal cracking and pore-pressure diffusion in fractured rock. Each anomaly type provides critical insight into reservoir properties.

Fault-related anomalies mark fracture zones and hydrothermal alteration, where porosity and permeability enhance steam and fluid circulation. These zones consistently appear as low Vs anomalies, providing reliable targets for microseismic monitoring of fracture activation. Their link to anisotropy and fluid flow highlights the value of mapping fractures to optimize production and reduce seismic risk. The second anomaly type relates to the steam cap above the NTR. Across the depth range of the NTR surface (~ 900–1400 m), sharp Vs transitions reflect steam saturation and strong thermal-fluid contrasts. Monitoring this boundary with time-lapse tomography captures the evolution of the steam cap and associated stress changes, offering crucial insights into reservoir stability. The third anomaly type stems from water injection, in which cold fluids alter hot fractured rocks via thermal cracking and pore-pressure diffusion, creating localized or vertically extended low Vs zones. Temporal variations of these anomalies reveal the reservoir’s response to injection cycles, while integration with microseismicity data constrains fracture activity, fluid migration, and saturation, supporting effective and sustainable monitoring.

In alignment with broader advances in geothermal technologies (including enhanced drilling methods, closed-loop systems, underground thermal energy storage, and advanced numerical modeling) the seismological framework presented here underscores the pivotal role of monitoring in ensuring long-term reservoir sustainability and operational efficiency. By linking structural anomalies with physical processes, our study demonstrates how seismology bridges scientific exploration and practical management. This integrated approach is crucial not only for optimizing geothermal production but also for addressing societal concerns related to seismic hazards, environmental impacts, and the sustainable deployment of geothermal energy as a reliable low-carbon resource.

## Supplementary Information

Below is the link to the electronic supplementary material.


Supplementary Material 1.


## Data Availability

The raw seismic signals used in this study are available from the NCEDC repository^16^ (https://www.ncedc.org/web-services-home.html; last accessed April 2026). Also, the processed dataset generated and analyzed during this study can be obtained from the corresponding author on reasonable request.
