# Supplementary material for: Imaging subsurface structures near wells in the northwest Geysers geothermal site
Source: Sci Rep. 2026 Jun 15;16:27328. doi: 10.1038/s41598-026-57720-x (PMC13530241; doi:10.1038/s41598-026-57720-x)
Supplement: Supplementary file 1 [file 41598_2026_57720_MOESM1_ESM.docx]

**Imaging Subsurface Structures Near Wells in the Northwest Geysers Geothermal Site**

**Taghi Shirzad^1^**, **Mohsen Kazemnia^2^**, **Beata Orlecka‐Sikora^3^**, **Götz Bokelmann^4^, Saman A. Aryana^5^**

^1^ Institute of Astronomy, Geophysics and Atmospheric Sciences, University of São Paulo, São Paulo, Brazil (05508-090). Email: *t.shirzad@iag.usp.br*; ORCID: 0000-0002-8382-4990

^2^ Department of Meteorology and Geophysics, University of Vienna, Vienna, Austria. Email: *mohsen.kazemnia.kakhki@univie.ac.at;* ORCID: 0000-0002-8519-6502

^3^ Institute of Geophysics, Polish Academy of Sciences, 01-452, Warsaw, Poland. Email: *orlecka@igf.edu.pl*; ORCID: 0000-0002-0182-1455

^4^ Department of Meteorology and Geophysics, University of Vienna, Vienna, Austria. Email: *Goetz.Bokelmann@univie.ac.at* ORCID: 0000-0001-5677-7677

^5^ Department of Chemical & Biomedical Engineering, University of Wyoming, Wyoming, USA. Email: Saryana@uwyo.edu

*Corresponding author*: Taghi Shirzad*

**Fig. S1** Phase velocity tomographic maps at period range of 0.05-0.2 s at depth of 1.1km. Known faults (Squaw Fault, Creek Fault, and Caldwell Pines Fault, C.P.F.) are shown as red lines. Injection and steam wells are indicated by squares. Injection wells are shown as blue squares and steam wells as purple squares.

**Fig. S1 (continued)** Phase velocity tomographic maps at period range of 0.05-0.2 s at depth of 1.2 km. Known faults (Squaw Fault, Creek Fault, and Caldwell Pines Fault, C.P.F.) are shown as red lines. Injection wells are shown as blue squares and steam wells as purple squares.

**Fig. S1 (continued)** Phase velocity tomographic maps at period range of 0.05-0.2 s at depth of 1.3 km. Known faults (Squaw Fault, Creek Fault, and Caldwell Pines Fault, C.P.F.) are shown as red lines. Injection wells are shown as blue squares and steam wells as purple squares.

**Fig. S1 (continued)** Phase velocity tomographic maps at period range of 0.05-0.2 s at depth of 1.4 km. Known faults (Squaw Fault, Creek Fault, and Caldwell Pines Fault, C.P.F.) are shown as red lines. Injection wells are shown as blue squares and steam wells as purple squares.

**Fig. S1 (continued)** Phase velocity tomographic maps at period range of 0.05-0.2 s at depth of 1.5 km. Known faults (Squaw Fault, Creek Fault, and Caldwell Pines Fault, C.P.F.) are shown as red lines. Injection wells are shown as blue squares and steam wells as purple squares.

**Fig. S1 (continued)** Phase velocity tomographic maps at period range of 0.05-0.2 s at depth of 1.6 km. Known faults (Squaw Fault, Creek Fault, and Caldwell Pines Fault, C.P.F.) are shown as red lines. Injection wells are shown as blue squares and steam wells as purple squares.

**Fig. S1 (continued)** Phase velocity tomographic maps at period range of 0.05-0.2 s at depth of 1.7 km. Known faults (Squaw Fault, Creek Fault, and Caldwell Pines Fault, C.P.F.) are shown as red lines. Injection wells are shown as blue squares and steam wells as purple squares.

**Fig. S1 (continued)** Phase velocity tomographic maps at period range of 0.05-0.2 s at depth of 1.8 km. Known faults (Squaw Fault, Creek Fault, and Caldwell Pines Fault, C.P.F.) are shown as red lines. Injection wells are shown as blue squares and steam wells as purple squares.

**Fig. S1 (continued)** Phase velocity tomographic maps at period range of 0.05-0.2 s at depth of 1.9 km. Known faults (Squaw Fault, Creek Fault, and Caldwell Pines Fault, C.P.F.) are shown as red lines. Injection wells are shown as blue squares and steam wells as purple squares.

**Fig. S1 (continued)** Phase velocity tomographic maps at period range of 0.05-0.2 s at depth of 2.0 km. Known faults (Squaw Fault, Creek Fault, and Caldwell Pines Fault, C.P.F.) are shown as red lines. Injection wells are shown as blue squares and steam wells as purple squares.
